# Integrated analysis of circulating and tissue proteomes reveals that fibronectin 1 is a potential biomarker in papillary thyroid cancer

**DOI:** 10.1186/s12885-023-10839-w

**Published:** 2023-05-08

**Authors:** Guochao Ye, Xiaomei Zhang, Mansheng Li, Zixiang Lin, Yongcan Xu, Haoru Dong, Jie Zhou, Jiaqi Zhang, Sheng Wang, Yunping Zhu, Xiaobo Yu, Xu Qian

**Affiliations:** 1 Department of General Surgery, Huzhou Central Hospital, Huzhou, 313000 P. R. China; 2 State Key Laboratory of Proteomics, Beijing Proteome Research Center, National Center for Protein Sciences-Beijing (PHOENIX Center), Beijing Institute of Lifeomics, Beijing, 102206 P. R. China; 3 School of Basic Medical Sciences, Peking University, Beijing, 100191 P. R. China; 4 Department of Clinical Laboratory, Zhejiang Cancer Hospital, Institute of Basic Medicine and Cancer (IBMC), The Chinese Academy of Sciences, Hangzhou, 310022 P. R. China

**Keywords:** Thyroid cancer proteome, Thyroid nodules, Indeterminate thyroid biopsy, Cancer secrotome, FN1

## Abstract

Papillary thyroid cancer (PTC) is the most frequent subtype of thyroid cancer, but 20% of cases are indeterminate (i.e., cannot be accurately diagnosed) based on preoperative cytology, which might lead to surgical removal of a normal thyroid gland. To address this concern, we performed an in-depth analysis of the serum proteomes of 26 PTC patients and 23 healthy controls using antibody microarrays and data-independent acquisition mass spectrometry (DIA-MS). We identified a total of 1091 serum proteins spanning 10–12 orders of magnitude. 166 differentially expressed proteins were identified that participate in complement activation, coagulation cascades, and platelet degranulation pathways. Furthermore, the analysis of serum proteomes before and after surgery indicated that the expression of proteins such as lactate dehydrogenase A and olfactory receptor family 52 subfamily B member 4, which participate in fibrin clot formation and extracellular matrix-receptor interaction pathways, were changed. Further analysis of the proteomes of PTC and neighboring tissues revealed integrin-mediated pathways with possible crosstalk between the tissue and circulating compartments. Among these cross-talk proteins, circulating fibronectin 1 (FN1), gelsolin (GSN) and UDP-glucose 4-epimerase (GALE) were indicated as promising biomarkers for PTC identification and validated in an independent cohort. In differentiating between patients with benign nodules or PTC, FN1 produced the best ELISA result (sensitivity = 96.89%, specificity = 91.67%). Overall, our results present proteomic landscapes of PTC before and after surgery as well as the crosstalk between tissue and the circulatory system, which is valuable to understand PTC pathology and improve PTC diagnostics in the future.

**Supplementary Information:**

The online version contains supplementary material available at 10.1186/s12885-023-10839-w.

## Background

Thyroid cancer is the ninth most common malignancy worldwide [1]. In China, continuous increases in thyroid cancer have been reported; the incidence increased from 2.75/10^5^ in 2000 to 19.42/10^5^ in 2012 in the Zhejiang Province of China [2, 3]. Papillary thyroid cancer (PTC) is the most prevalent form of thyroid cancer and therefore contributes the rapid increase in thyroid cancer [2, 4]. Oversurveillance and overdiagnosis of smaller differentiated cancers are attributed to increased incidence and consequently accompanied by overtreatment [5]. Efforts are urgently needed to prevent overtreatment of low-risk papillary thyroid cancers with the aim of improving quality of life and reducing the economic burden [5, 6]. Notably, there are limitations to preoperative cytology by fine-needle aspiration (FNA) biopsy, as 20% of cases are diagnosed as indeterminate [7]. Moreover, the majority of indeterminate cases are found to be benign nodules based on histopathology after surgery [7]. There are potential risks and side effects associated with overtreatment such as hypoparathyroidism and the need for lifelong thyroid hormone (levothyroxine) replacement therapy after total thyroidectomy. Therefore, there is an unmet need to identify which thyroid nodules are benign and do not require the FNA or surgery.

Molecular testing, such as that for the *BRAF V600E* mutation, has been suggested to distinguish aggressive PTCs; the *BRAF V600E* mutation accounts for 60% of mutations in thyroid cancer [8]. Recent studies have shown that combining the BRAF V600E mutation and the Bethesda System for Reporting Thyroid Cytopathology increased sensitivity (89.57%) for malignant nodules and the negative predictive value (45.45%) for benign nodules in patients who underwent ultrasound-guided FNA [9]. However, the genotyping of PTCs has not been established in routine practice and needs further longitudinal large cohort studies.

We hypothesized that the incorporation of circulating tumor-specific functional proteomic markers holds promise to help determine a suspicious nodule. Previously, proteomics-based approaches have successfully identified subtypes of certain cancers, including gastric cancer, hepatocellular carcinoma and lung adenocarcinoma [10–12]. Studies of PTC tissues and FNA samples have demonstrated that a spectrum of proteins or metabolites in thyroid tissue has the potential to differentiate malignant thyroid nodules [13, 14]. Although these candidate tissue biomarkers warrant further clinical validation, developing a minimally invasive test that allows blood-based biomarker discovery for early diagnosis of PTC and treatment monitoring would also be important. Ideally, secretomes may temporally reflect the status of diseased and healthy cells. To test this hypothesis, we used an in-depth serum proteomics platform including customizable antibody microarrays and data independent acquisition mass spectrometry (DIA-MS) to detect the differential serum protein expression in paired serum and tumor tissues and their paired noncancerous adjacent tissues (NATs) for the same patient with PTC [15]. This platform enables us to detect low-abundance proteins by spanning 10 orders of magnitude in protein concentration. We demonstrate crosstalk between the serum and tissue proteomes and provide new insights into the oncobiology of PTC. We conclude from these findings that the relatively noninvasive assay of these candidate circulating proteins, which could be performed frequently, may aid the clinical decision-making process and warrant further clinical validation for predicting malignancy.

## Methods

### Clinical samples and patient characteristics

Fresh frozen cancer tissues, paired NATs and serum samples before and after thyroidectomy were obtained from 26 patients with PTC. Serum samples from 23 healthy controls (HCs) were also obtained. The patients and HCs participating in this study all signed an institutional review board-approved informed consent form. This study was approved by the local Ethics Committee of Zhejiang Cancer Hospital (#IRB-2020–371) and Huzhou Central Hospital (#IRB-20180804–01). The patients and HCs were similar in age with a mean age of 39.7 ± 12.8 years. The eighth edition of the tumor/lymph node/metastasis (TNM) staging system of the American Joint Committee on Cancer was used to assign the stage of all patients. Most PTC patients were staged as stage I (25 of 26) with a TI-RADS Score 6 (25 of 26) (Table 1, Additional file 1: Table S1-1). The BRAF V600E mutation was found in 13 patients. Our validation cohort included serum samples from 36 patients with benign nodules and 61 patients with PTC (data not shown).

**Table 1:** Clinicopathological characteristics of patients with papillary thyroid cancer (*n* = 26)

Variables	N	%
Age, y, mean ± SD (range)	26	39.7 ± 12.8
Sex		
Male	7	26.9
Female	19	73.1
T stage		
T1/T2	21	80.8
T3/T4	5	19.2
LNM		
N0	14	53.8
N1	12	46.2
TNM stage		
I	25	96.2
III	1	3.8
TI-RADS Score		
4	1	3.8
6	25	96.2
BRAF^V600E^		
Mutant	13	50.0
Wild	2	7.7
Unknown	11	42.3

### Screening of the serum proteome using antibody microarrays

The antibody microarrays were prepared as previously described [15]. Ten microliters of serum was diluted 1:10 with phosphate buffered saline (PBS; pH 7.4) and then labeled with NHS-PEG4-Biotin (Thermo Fisher Scientific, MA, USA). After removing the excess biotin molecules, the biotinylated serum was diluted with 400 μL of 5% milk (w/v) and then incubated with antibody microarrays that were blocked for 1 h at room temperature with 500 μL of 5% milk (w/v). Subsequently, the antibody microarrays were washed with PBS containing 0.05% (w/v) Tween 20 (PBST). The bound proteins on microarrays were detected by incubating with 2 µg/mL streptavidin–phycoerythrin (PE) (Jackson Immunoresearch, USA) for 1 h at room temperature. After washing and drying, the microarrays were scanned using the GenePix 4300A microarray scanner.

### Measurement of serum and tissue proteomes using DIA-MS

Protein separation, peptide sample preparation and DIA analysis were performed as previously described [16]. Briefly, total protein was extracted from tumor tissues and their paired NATs using RIPA buffer containing 10% protease inhibitor cocktail. The protein concentration was quantified by the Bradford method. The serum samples were diluted with lysis buffer containing 6 M urea (Sigma, USA). Next, extracted tissue protein and diluted serum were reduced with 10 mM dithiothreitol (DTT) at 37 °C for 60 min. After alkylating with 50 mM iodoacetamide (IAA) at room temperature for 45 min in the dark, the protein was digested with trypsin. The concentrations of tryptic peptides were determined by absorbance measurements with NanoDrop spectrophotometers (Thermo Scientific, USA). For construction of the spectral library, 10 μg of peptide pool from each sample was separated into 10 fractions, and data-dependent acquisition (DDA) analysis was performed on a QE-HF mass spectrometer (Q Exactive HF Hybrid Quadrupole Orbitrap, Thermo Fisher). A human subset of the UniProt proteins FASTA database was used to generate a spectral library using Spectronaut Pulsar X 12.0 (Biognosys, Schlieren, Switzerland) with the BGS factory setting. A 1% protein false discovery rate (FDR) and at least 2 peptides per protein were considered confident identification. For DIA analysis, 1.5 µg peptides were separated on a 30 min LC gradient using an analytical column (150 µm × 250 mm; 2 µm, 200 Å C18 particles) and analyzed by mass spectrometry as described above. The DIA acquisition scheme consisted of 45 fixed windows ranging from 350 to 1500 m/z. The resolution distributions of MS1 and MS2 were 60,000 and 30,000, respectively. The raw files were input into Spectronaut software for analysis using the default settings with quantification on the MS2 level of the top N (1–3) peptide spectra, and the results were filtered by a 1% FDR.

### RNA-seq analysis

Gene count data (RNA-seq) of thyroid carcinoma samples were downloaded from The Cancer Genome Atlas database (TCGA; https://portal.gdc.cancer.gov/repository). The annotation information was downloaded from the GENCODE (GRCh38.p13) catalog (https://www.gencodegenes.org/). Averaged values were taken for multiple probes sharing the same gene. Next, the “edgeR” package was utilized to perform differential expression analysis (adjusted *P* value < 0.05 and |Log (fold change)|> 1). After standardizing the quantified data with the “scale” function, the heatmap was generated by using the “pheatmap” package. Twenty-nine differentially expressed genes, consistent with the results of proteomic analysis, are shown in the heatmap. Gene Ontology (GO) and Kyoto Encyclopedia of Genes and Genomes (KEGG) analyses were conducted using the “clusterProfiler”, “org.Hs.eg.db”, and “enrichplot” packages in R to provide functional annotation and analyze pathway enrichment [17]. An adjusted *P* < 0.05 was considered statistically significant. GO enrichment analysis included the molecular function (MF), biological process (BP), and cellular component (CC) categories. The top 15 KEGG pathways and top 5 GO terms were visualized using the “GOplot” package.

### Validation of biomarker candidates by IHC and ELISA

After deparaffinization in xylene, hydration with graded alcohol, and antigen retrieval, the tissue sections were placed in 3% hydrogen peroxide (H_2_O_2_) for 10 min at room temperature to inactivate endogenous peroxidases. The slides were washed three times in phosphate-buffered saline (PBS), blocked with 2% bovine serum albumin (BSA) for 30 min at room temperature and incubated with primary antibodies at 4 °C overnight. On the second day, after washing with PBS, the slides were incubated with HRP-conjugated secondary antibodies for 60 min at 37 °C. The slides were then washed in PBS followed by detection with DAB staining solution and counterstaining with hematoxylin.

The levels of gelsolin and fibronectin in the serum of the different groups were measured by their respective enzyme-linked immunosorbent assay (ELISA) kits. The optical density of each sample was measured at 450 nm using a Thermo Scientific Varioskan Flash Spectral Scanning Multimode Reader.

### Statistical and bioinformatics analysis

For the antibody-array data, the intensities of identified proteins were corrected by subtracting the background signal, and the values were then log2 transformed and interarray normalized with quantile normalization followed by mean centering. After background correction and signal normalization, the intensities could be used for the downstream analysis. For the MS data, the quantification values of identified proteins were log2 transformed and mean normalized (Figure S1).

To test for significant differences in the expression of proteins between serum samples from healthy controls and PTC patients before and after thyroidectomy, multiple comparisons were performed with the R package Limma (V3.38.3) [18]. The differentially expressed serum proteins between the two groups were analyzed using Limma with a *P* value < 0.05. To identify differential proteins in tumor versus NAT, a modified t test was applied to tissue proteomic data. The *P* values were then adjusted by the Benjamini‒Hochberg method. Proteins with an adjusted *P* value < 0.05 were considered to be statistically significant.

Volcano plots and heatmaps of significant proteins were generated using the R packages “ggplot2” and “ComplexHeatmap” (distance, Pearson; linkage, complete) [19]. The distribution of serological protein concentrations was detected by our DIA-MS and microarray platform using the reference concentrations from the human plasma proteome database (http://www.plasmaproteomedatabase.org/). The online tool DAVID (https://david.ncifcrf.gov/) was used to annotate proteins according to GO and KEGG pathway analyses [20]. The protein interactome network was built using Cytoscape (version 3.7.1) [21], and the protein‒protein interactions were retrieved from the STRING database [22]. The common differentially expressed proteins from serum and tissue were subjected to in-depth analysis based on ingenuity pathway analysis (IPA).

The area under a ROC curve and graphs of the true positive rate (sensitivity) and the false-positive rate (specificity) were used to determine the diagnostic accuracy of the ELISA test.

### Random forest-based machine learning model

Based on the proteomic data of serum and tissue samples, we developed a new random forest (RF)-based classifier to identify potential biomarkers and classify HCs cases (Figure S2A). The computational pipeline development contains the following four steps: (i) dataset preprocessing, (ii) feature selection, (iii) model training, and (iv) model evaluation.

The log2-transformed and mean-normalized data from healthy and PTC patient serum samples and the tumor tissues and their paired NAT samples were retained. We then used the R package “caret” (v.6.0–93) to sample 70% of the serum cohort (HCs, *n* = 26; PTC, *n* = 22) and tissue cohort (NAT, *n* = 21; tumor, *n* = 21) as a training set; the remaining samples of the serum cohort and tissue cohort were used as an independent testing set. The codifferentially expressed proteins in serum and tissue were selected as features to distinguish HCs from PTC tumors. We tuned the parameters using a grid search algorithm with threefold cross-validation implemented in the “caret” package. We then retrained the RF model using the optimal parameters. Finally, the performances of the RF models were further evaluated based on the independent testing sets. The corresponding confusion matrices and receiver operating characteristic (ROC) plots were generated to assess performances using the “caret”, “plotROC” (v.2.2.1), and “ggplot2” packages.

## Results

### Serum proteomic analysis shows dysregulation in multiple biological processes in PTC patients

**Fig. 1:**
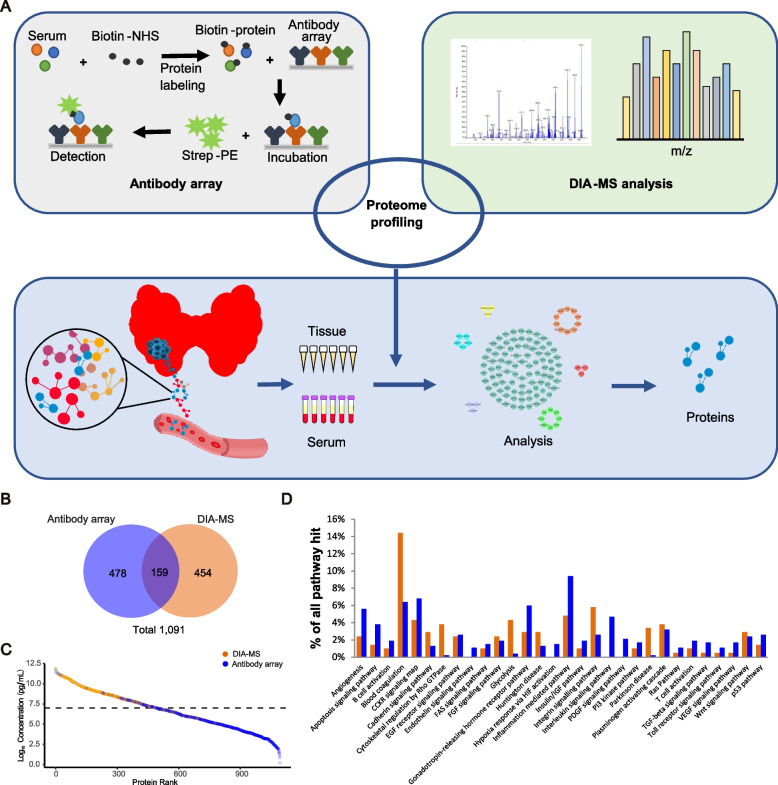
Study design using in-depth serum proteomics and tissue proteomics of papillary thyroid cancer (PTC). **A** work flow. **B** a total of 1091 serum proteins with an overlap of 159 proteins was detected through customizable antibody microarrays and DIA-MS. **C** Distribution of serum proteins detected by DIA-MS and antibody microarray two-pronged approach. **D** Proteins acquired from antibody microarrays and DIA-MS are belonged to the same cellular components

The workflows of in-depth serum proteomics and tissue proteomics of papillary thyroid cancer are shown in Fig. 1A. The antibody microarray detected 637 proteins, and DIA-MS detected 613 proteins for a total of 1091 serum proteins with an overlap of 159 proteins by both platforms (Fig. 1B, Additional file 2: Table S2-1, Table S2-2) distributed across approximately 10 orders of magnitude of abundance in plasma (Fig. 1C). Quality control demonstrated that the *r* correlations for array-to-array and slide-to-slide were 0.70 and 0.94, respectively, for antibody microarrays (Supplementary Fig. 1). The Pearson correlation analysis demonstrated a clear grouping of proteins among all samples acquired by DIA-MS (Fig. 1, Supplementary Fig. 1). Proteins acquired from antibody microarrays and DIA-MS belong to the same cellular components (Fig. 1D). In particular, a spectrum of proteins was associated with signaling pathways such as integrin signaling pathways, angiogenesis, blood coagulation, glycolysis, inflammation mediated pathway, interleukin and the CCKR-pathway (Fig. 1D).

**Fig. 2:**
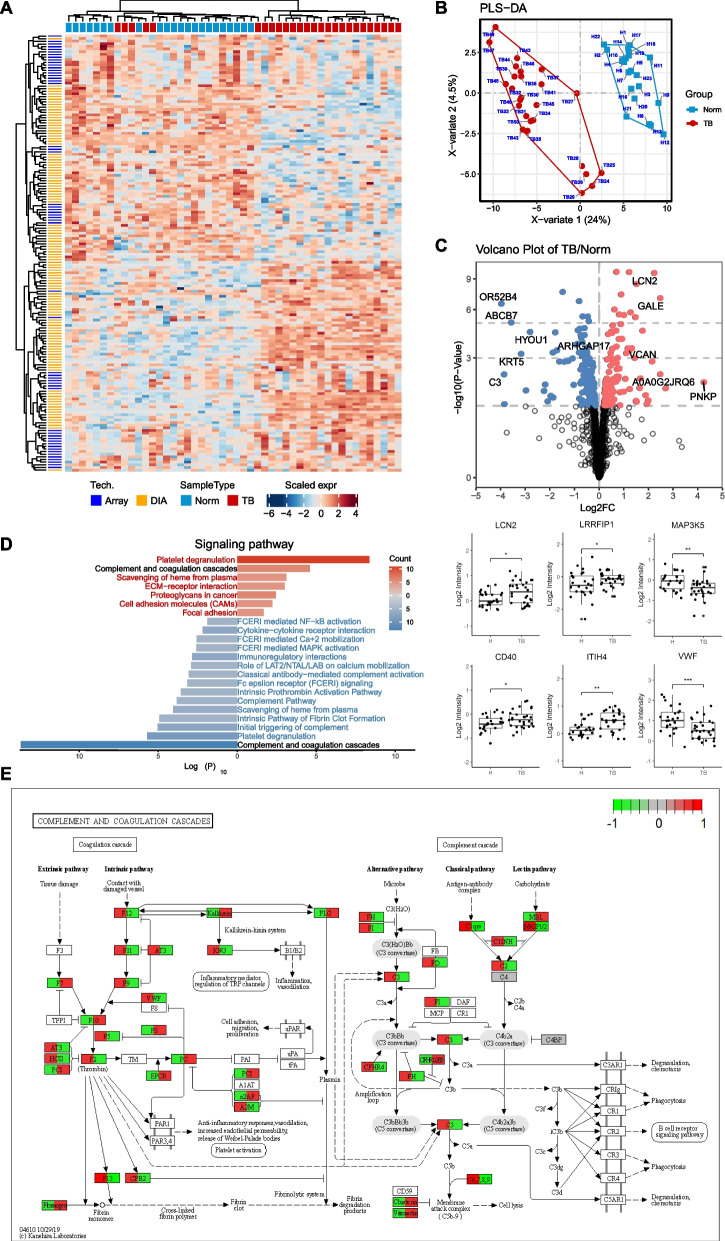
Serum proteome detection consisting of healthy controls (HCs) and patients with papillary thyroid cancer (PTC). **A** Classification of HCs and PTC patient groups based on differentially expressed proteins and unbiased clustering analysis. **B** Scores plot for Partial least square-discriminant analysis (PLS-DA). Score plot shows TB in red and controls in blue polygon. **C** Identification of PTC-associated proteins in serum using volcano plot analysis. Box plot analysis of represented PTC-associated serum proteins. **D** are the comparison of protein classes and signaling pathways for PTC-associated proteins and HCs. **E** The complement cascade

We first sought to define the serum proteomics of PTC patients and HCs. We found 166 significantly differentially expressed proteins between the two groups (*P* value < 0.05) (Fig. 2, Additional file 3: Table S3) of which 54 proteins were detected by antibody microarray, 117 were detected by DIA-MS, and 5 were detected by both antibody microarray and DIA-MS. For the differentially expressed proteins that could be detected by both methods, those with high significance were retained for further analysis. These dysregulated proteins mainly belonged to the complement and coagulation cascades and platelet degranulation pathways (Fig. 2D, E). In addition, PTC patients had activated pathways, including extracellular matrix (ECM)-receptor interactions, proteoglycans, cell adhesion molecules and focal adhesion, whereas the high-affinity immunoglobulin E receptor (FcεRI)-mediated signaling pathways were downregulated compared with normal controls (Fig. 2D). Notably, increased serum C3 and deceased serum apolipoprotein A4 in PTC patients were shown in our study and in other studies using mass spectrometry and ELISA validation [23].

**Fig. 3:**
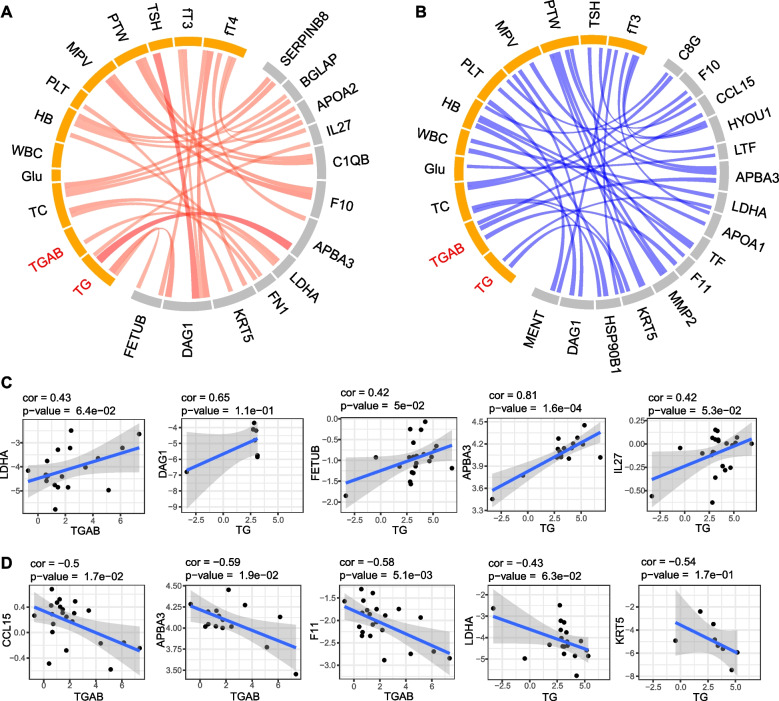
Correlation network of serum proteome and clinical data. **A** Positive and (**B**) negative correlations between serum proteome and clinical data using circus, respectively. Reprehensive examples for positive (**C**) and (**D**) negative correlations

We further analyzed the data to identify the association between proteomic changes and clinical data (Fig. 3). Pearson correlation analysis demonstrated that APBA3 was positively correlated with serum levels of thyroglobulin (Tg) and negatively correlated with serum levels of Tg antibodies (TgAb). Tg is an iodoglycoprotein made by thyroid cells and serves as a precursor for triiodothyronine (T3) and thyroxine (T4) hormones. Although serum levels of Tg cannot predict disease stage for PTC, small thyroid remnants can be detected by serum levels of Tg during follow-up after total thyroidectomy [24]. However, following lobectomy in patients with thyroid cancer, Tg levels are influenced by lobe size, TSH levels, lymphocytic thyroiditis, thyroid nodules and other factors. It is challenging to detect Tg levels produced by persistent/recurrent cancer tissue when the total amount of Tg produced by the remaining lobe is measured. Thus, there is an unmet need for identifying other indicators for persistent cancer tissue and disease recurrence with regard to Tg dynamics. APBA3 is an activator of hypoxia inducible Factor 1 (HIF-1) and can mediate metastasis niche formation by recruiting monocytes and inducing E-selectin in endothelial cells [25]. Its correlation with Tg and the underlying mechanism in thyroid cancer warrant further investigation.

**Fig. 4:**
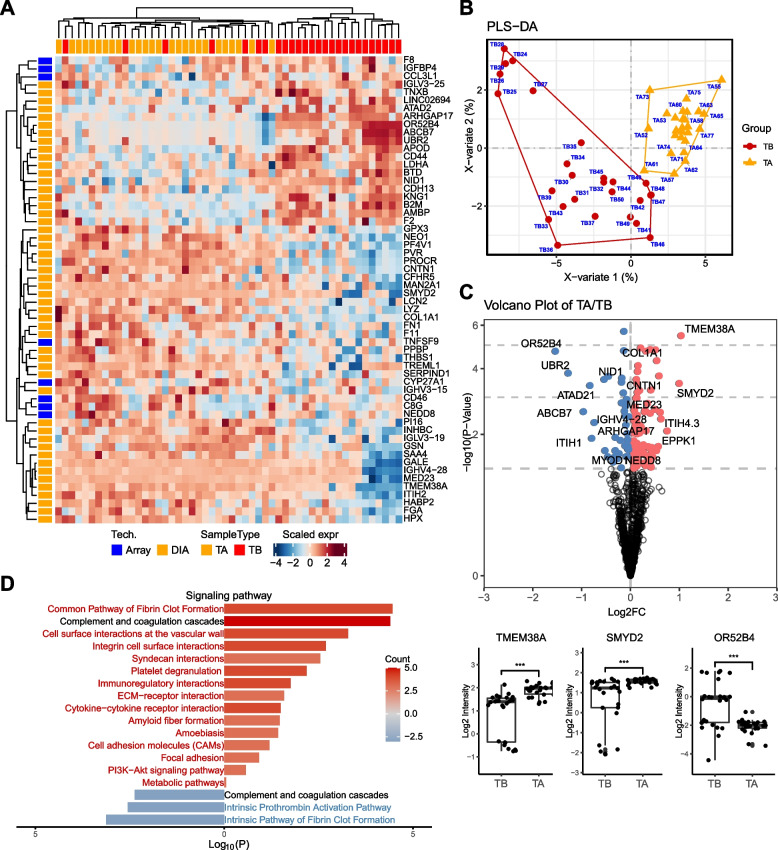
Serum proteome detection consisting of serums before and after surgery for the same patient with papillary thyroid cancer (PTC). **A** Classification of PTC patient before and after surgery based on differentially expressed proteins and unbiased clustering analysis. **B** Scores plot for Partial least square-discriminant analysis (PLS-DA). Score plot shows TB in red and TA in blue polygon. **C** Identification of differentiated proteins in serum of PTC patient before and after surgery using volcano plot analysis. Box plot analysis of represented serum proteins. **D** are the comparison of protein classes and signaling pathways for PTC patient before and after surgery

There is substantial interest in the potential of proteomic profiling to aid in risk stratification after surgery and to optimize treatment decisions. We identified an additional 58 significantly differentially expressed serum proteins between PTC patients before and after tumor removal (Fig. 4). We found that dysregulation of complement and coagulation cascades are the main pathway differences between the two states. The intrinsic pathway of fibrin clot formation and the intrinsic prothrombin activation pathway were activated in primary PTC patients without treatment, and pathways such as the intrinsic pathway of fibrin clot formation and ECM-receptor interaction were downregulated after tumor removal. Notably, the serum level of lactate dehydrogenase A (LDHA) was decreased after tumor removal. This may reflect the change in tumor burden, as seen in a previous study showing that LDHA is overexpressed in PTC tissue and represents aggressive PTC behavior [26]. LDHA functions as an enzyme that converts pyruvate to lactate in the final step of glycolysis. These findings warrant further investigation in the follow-up of PTC patients in an independent cohort.

### Proteomic features in PTC tumor tissues compared with NATs

**Fig. 5:**
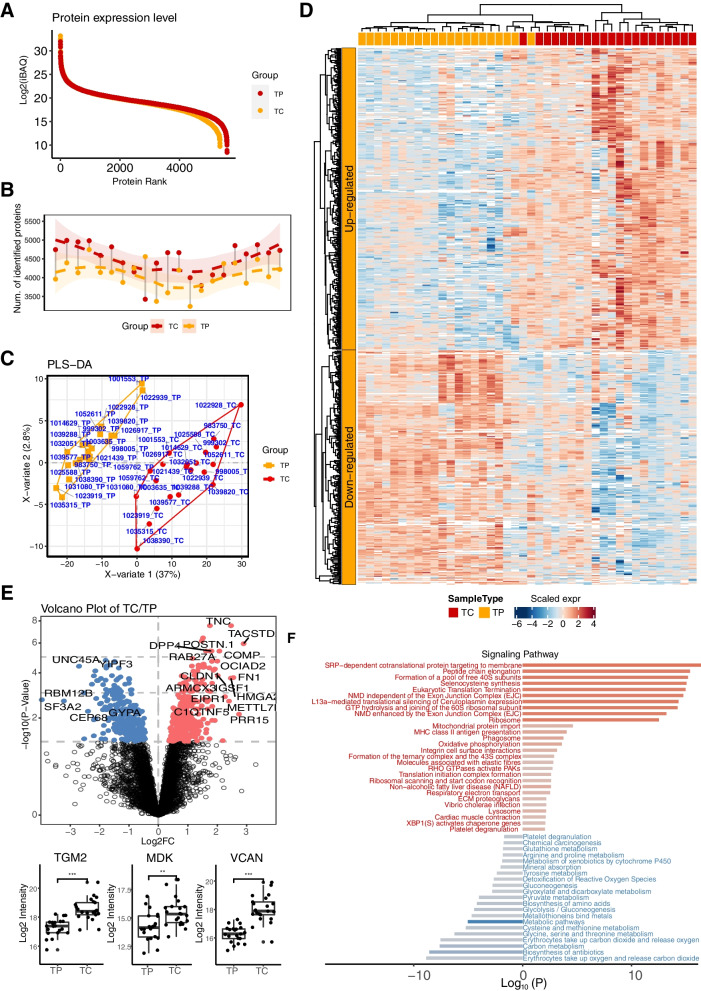
Tissue proteome detection consisting of tumor tissues (labeled as TC) and their paired non-cancerous adjacent tissues (NATs) (labeled as TP) for the same patient with papillary thyroid cancer (PTC). **A** The average intensity of the identified proteins was plotted with rank to illustrate the dynamic range of the tissue proteome. Points in red are the proteins from TC sample and points in orange are the proteins form TP. **B** Overview of the protein identifications in TC samples. The pairwise samples are annotated by grey straight lines. The dashed curves fitted by lasso regression show the distribution of protein identifications in TC (red) and TP (orange) samples. The shading that underlies the las-so curves denotes the 95% confidence intervals. **C** Scores plot for Partial least square-discriminant analysis (PLS-DA). Score plot shows TC in red and TP in blue polygon. **D** Classification of tumor tissues and their paired NATs based on differentially expressed proteins and unbiased clustering analysis. **E** Identification of differentiated proteins in tumor tissues and their paired NATs using volcano plot analysis. Box plot analysis of represented proteins. **F** are the comparison of protein classes and signaling pathways for tumor tissues and their paired NATs

We compared tumors vs. paired NATs to identify PTC-associated alterations in proteins. A total of 5648 total quantified proteins were identified, and 4826 proteins were included in the final analysis after filtering with the missing value (Additional file 4: Table S4). Differential protein expression analysis resulted in 612 significantly dysregulated proteins between PTC tissues and NATs, which accounted for 12.7% of the total quantified tissue proteome (Fig. 5, Additional file 5: Table S5). Among these, 344 were upregulated and 268 were downregulated in PTC tumor tissues. The representative proteins are shown in Fig. 5D. Notably, the number of proteins identified in the PTC tissues was significantly higher than that identified in the NATs. This finding is consistent with the common enriched pathways seen in the functional pathway annotation and enrichment analysis of the dysregulated PTC proteomes, which revealed that tissue-specific biological networks belonged to protein translation and the immune system. Specifically, proteins were involved in signal recognition particle (SRP)-dependent cotranslational protein targeting to the membrane, peptide chain elongation, MHC class II antigen presentation and metabolism-related pathways (Fig. 5F). For example, SRP is essential for delivering the proteome to the proper cellular membrane as integral membrane proteins or secretions [27].

**Fig. 6:**
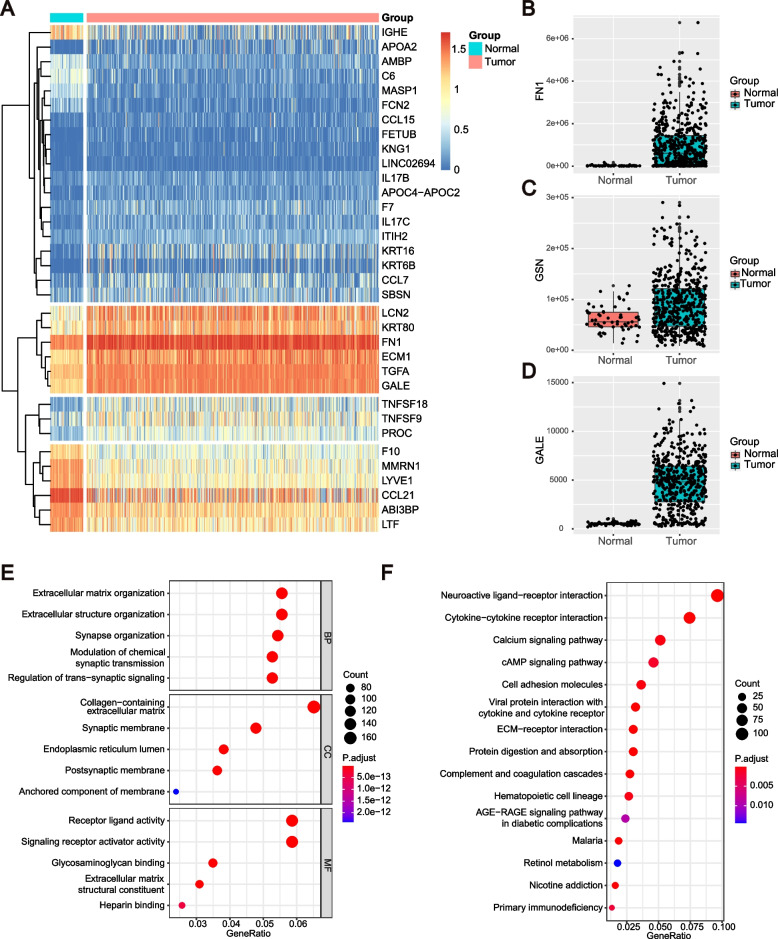
Characterization of the transcriptomes data of patients with papillary thyroid cancer. **A** The heatmap of 34 selected genes in TCGA database. **B**-**D**, The boxplots indicate the genes expression levels between Normal and Tumor group. B represents FN1, C represents GSN and D represents GALE, respectively. Red boxplots refer to normal group, while green boxplots are for tumor group. **E**, **F** The results of functional enrichment analysis. Size of the dots represent the number of enriched genes, and the color of the dots represent the adjusted *P* value in GO (**E**) and KEGG (**F**) analyses

We next compared the represented tumor-enriched proteins at the transcriptional level with TCGA data from PTC tumor and paracarinoma transcriptomes (Fig. 6). The expression data of 568 thyroid carcinoma samples, including 510 tumor tissues and 58 paracarinoma tissues, were downloaded from TCGA. After differential expression analysis, 2781 differentially expressed genes (DEGs) were screened, and 29 DEGs, consistent with the results of proteomic analysis, are shown in the heatmap. The results of GO and KEGG pathways showed 2781 DEGs were involved in neuroactive ligand‒receptor interaction, cytokine‒cytokine receptor interaction, complement and coagulation cascades, ECM-receptor interaction and organization, collagen-containing extracellular matrix and receptor ligand activity, and tyrosine metabolism. Our analysis indicated similar clusters at the transcriptional level and protein level.

### Integration of the blood-thyroid proteome reveals an integrin-mediated signature in blood-tumor crosstalk

**Fig. 7:**
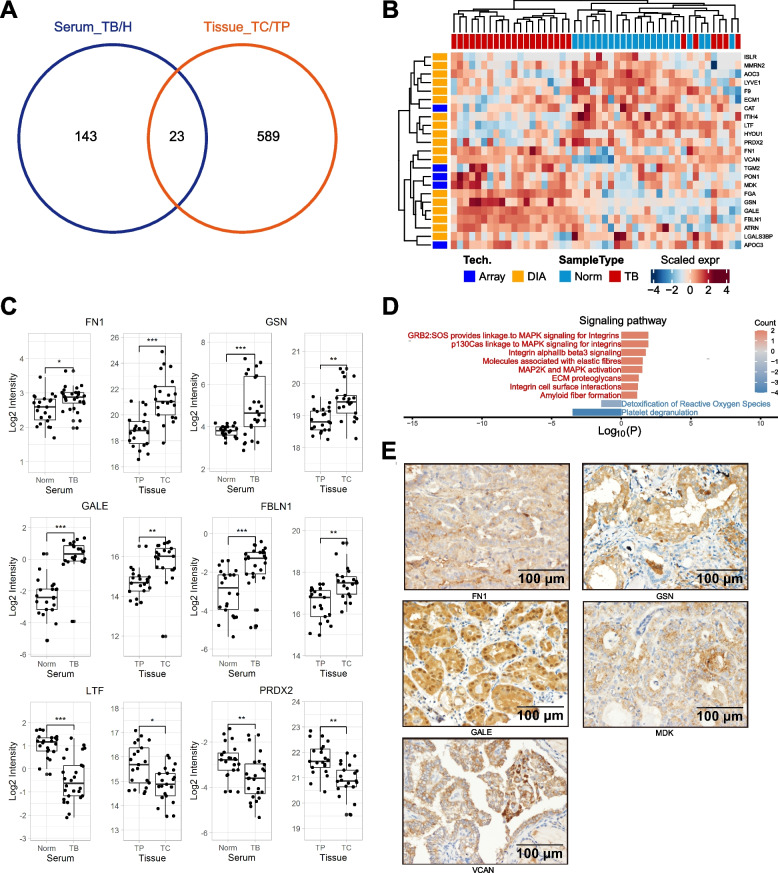
Characterization and validation of the integrated serum and tissue proteomics of patients with papillary thyroid cancer (PTC). **A** the overlapping 23 proteins presented in both serum and thyroid tumor tissue proteomic dataset. **B** Classification of integrated serum and tissue proteomics based on differentially expressed proteins and unbiased clustering analysis. **C** Differential expressions of represented proteins by DIA-MS and Antibody microarray. **D** are the comparison of protein classes and signaling pathways for 23 overlapped proteins. E, IHC demonstrates represented proteins expressions in PTC tissues

**Fig. 8:**
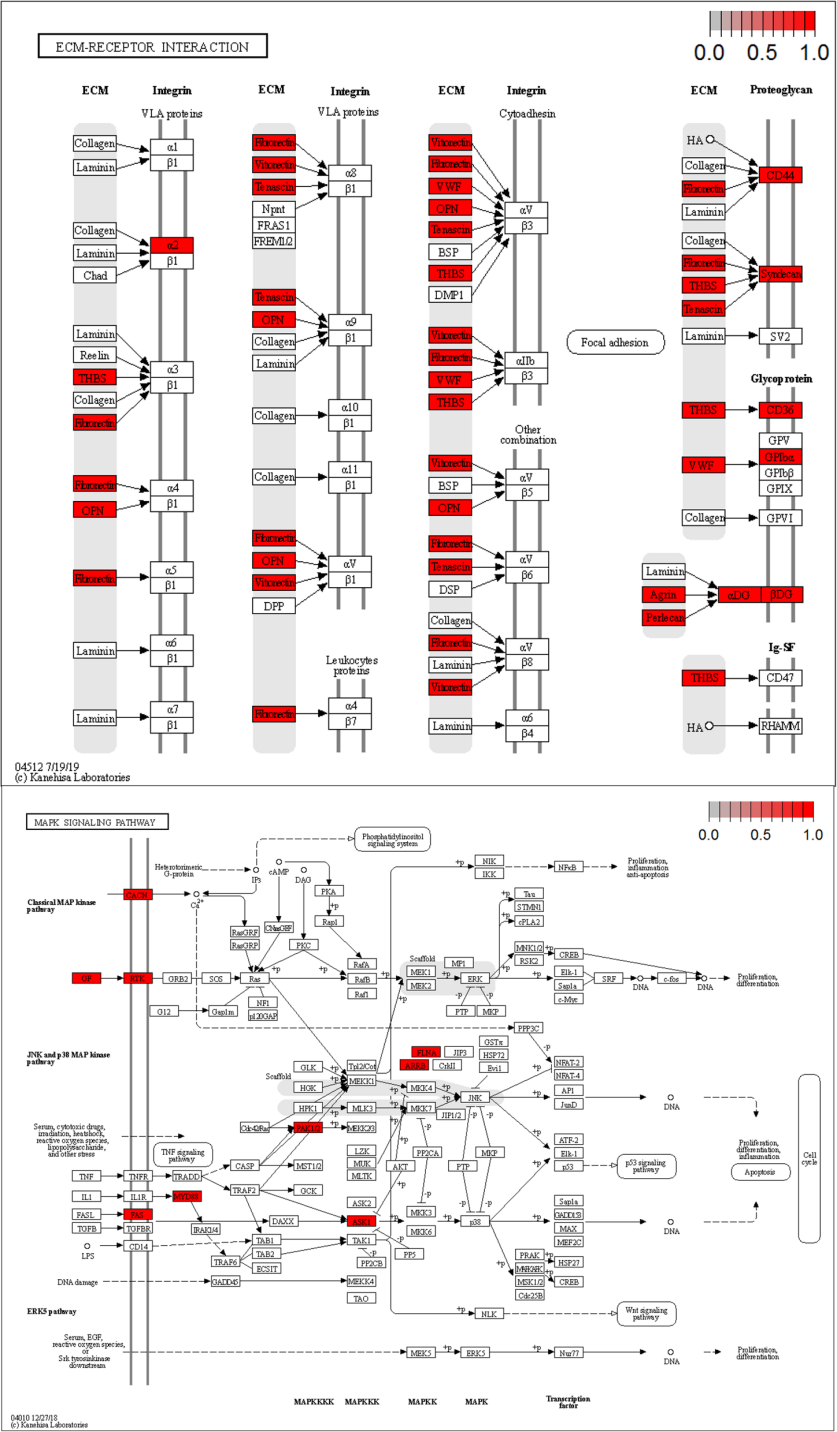
Extracellular matrix (ECM)-receptor interaction and MAPK pathway

**Fig. 9:**
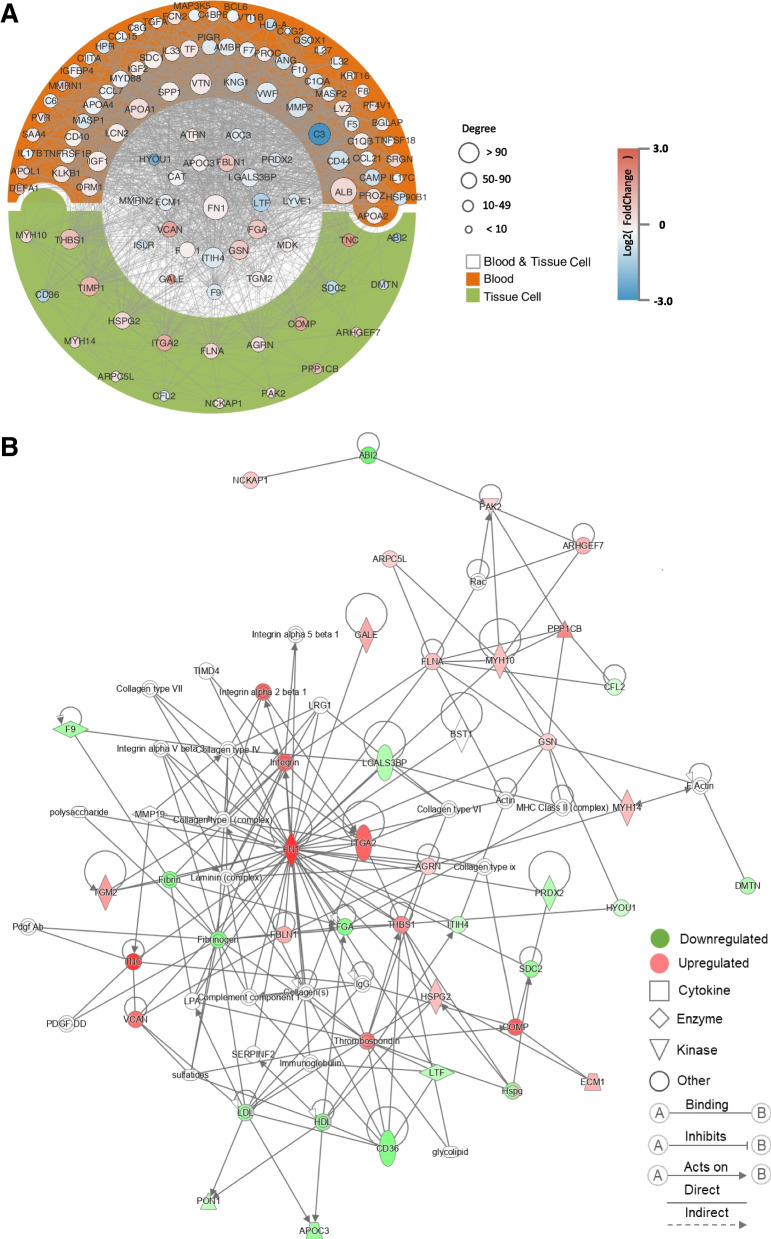
Networking and cross-talk of the integrated serum and tissue proteomics of patients with papillary thyroid cancer. **A** Identified protein interaction between blood and cancer tissue. **B** Ingenuity Path-way Analysis (IPA) Networks of Proteins. Red and green nodes indicate upregulated and downregulated molecules, respectively

We analyzed the overlapping 23 proteins present in both the serum and thyroid tumor tissue proteomic datasets (Fig. 7). To gain insight into the biological pathways related to molecular signals that may mediate blood-tumor tissue crosstalk, we used the GO term finder to classify the pathways. We found that mitogen-activated protein kinase (MAPK) signaling for integrins and ECM proteoglycans was upregulated in PTC patients. Integrins are a family of transmembrane glycoprotein signaling receptors, and their subunits can be found in platelets and tumor cells [28, 29]. Notably, the extracellular domain of integrin αvβ3 on the plasma membrane of tumor cells contains the receptor for thyroid hormone analogs [30]. It has been demonstrated that L-thyroxine (T4) binds the cell surface receptor on integrin αvβ3 and subsequently regulates cancer cell proliferation, angiogenesis and metastasis via the MAPK (ERK1 and ERK2 cascade) pathway [30]. Additionally, the BRAFV600E mutation is a risk factor for PTC and is related to poor clinical outcome [31]. It has been demonstrated that the BRAF mutation can activate the MAPK pathway, which is reversed by MAPK pathway inhibitors such as the mitogen-activated protein kinase inhibitor trametinib [32]. We identified 13 of 26 patients harboring the BRAFV600E mutation. In addition, platelet-tumor cell interactions have been proposed to participate in tumor metastasis [29]. Figures 8 and 9 demonstrates an IPA network of integrin-mediated pathways indicating the potential protein‒protein interactions in PTC. It would be interesting to further research T4 hormone-platelet/tumor cells via integrin signaling in a PTC model.

### Potential biomarkers that can distinguish PTC tumors from HCs

We built an RF-based classification model based on the 23 overlapping proteins presented in both the serum and thyroid tumor tissue proteomic datasets. Using these proteins and the RF algorithm, we obtained a mean AUC of the ROC curves of 0.990 on the combination training set of serum and tissue cohorts with the cross-validation scheme (Supplementary Fig. 2B-C). For the test sets, we obtained a mean AUC of the ROC curves of 1.000, 0.974 and 0.972 on the serum, tissue and combination cohort, respectively (Supplementary Fig. 2D). When we trained the model on one of the cohorts and tested it on the other, we obtained AUCs of 0.944 or 0.857 (Supplementary Fig. 2E). The results show that the 23 overlapping proteins have considerable potential to distinguish PTC tumors from HCs.

### Serum fibronectin 1, gelsolin and UDP-glucose 4-epimerase have the potential to differentiate patients with benign nodules and PTC

Specifically, 23 overlapping proteins were significantly correlated between PTC tissue and serum across patients. We observed higher expressions of fibronectin 1 (FN1), gelsolin (GSN) and UDP-glucose 4-epimerase (GALE) at the circulating and tissue levels of PTC patients compared with controls, as acquired by antibody microarrays and DIA-MS. We also observed high expressions of FN1, GSN and GALE in PTC tissue compared with normal tissues based on IHC (Fig. 7E). The mRNA levels of FN1, GSN and GALE were also highly expressed in PTC tissues based on RNA-seq in the TCGA dataset (Fig. 6 B-D).

We further observed that serum levels of FN1 and GSN were higher in PTC patients than in patients with benign nodules in an independent cohort based on ELISA. The results showed that the expression of FN1 and GSN were significantly higher in the PTC group than in the control group patients (*p* < 0.05, Supplementary Fig. 3A). The AUC of the FN1 biomarker was 0.9224, with a sensitivity of 96.89% and a specificity of 91.67%. The AUC of GSN was 0.637, with a sensitivity of 83.33% and a limited specificity of 50% (Supplementary Fig. 3B, Table 2). FN1, a glycoprotein, is an important component of the extracellular matrix. The biological role of FN1, particularly FN1-beta1 integrin signaling, in cancer progression, metastasis and therapy resistance has been explored in cancers such as lung cancer and breast cancer. GSN is a cytoskeletal protein that can promote epithelial-to-mesenchymal transition (EMT) signaling and subsequent tumor invasion. GALE is a glycosyltransferase involved in galactose metabolism. It has been shown that the mRNA levels of GALE and FN1 were overexpressed in PTC compared with benign nodules as acquired by RT-PCR [33]. These observations support a potential role for serum FN1, GSN and GALE in differentiating patients with benign nodules versus PTC. We did not validate GALE and other proteins due to the lack of commercial ELISA kits. It would be interesting to further validate them as potential biomarkers independently or in combination for PTC differential diagnostics in a large cohort.

**Table 2:** Area under the curve (AUC) and its 95% confidence interval (CI), sensitivity and specificity values of the ELISA tests

	FN1	GSN
AUC (95% CI)	0.924 (0.867–0.98)	0.637 (0.493–0.78)
Sensitivity (%)	86.89	83.33
Specificity (%)	91.67	50

## Discussion

Blood-based liquid biopsy has shown advantages in clinical settings as a minimally invasive, safe, and alternative or complementary approach for tissue biopsies. Importantly, tumor-derived secretomes or cancer degradomes in the TME play central roles in tumor progression, recurrence and metastasis [34]. Although DNA or RNA sequence data have been utilized to guide cancer treatment, a recent study demonstrated that the cancer proteome complements the DNA/RNA status and has the potential to refine treatment options [35]. In addition, proteome-wide depiction of interactomes across cancer and host responses at the tissue and circulating levels remains elusive. Here, we performed a comprehensive analysis of tissue and serum proteomes from PTC patients and healthy controls. We applied a strategy employing antibody microarrays and DIA-MS to quantify a total of 1091 serum proteins, which resulted in a much improved depth of the serum proteome.

The proteomic data from both serum and tumor tissue in PTC patients allowed us to identify the potential crosstalk between them. Our analysis demonstrates that the EMT markers FN1, GSN and GALE are strongly expressed in PTC tissues and at the circulating level. We further showed higher expression levels of FN1, GSN and GALE in PTC tissues than those in NATs. This finding is consistent with the transcriptional levels of FN1, GSN and GALE expression in the TCGA dataset of thyroid cancer. In particular, integrin αvβ3 contains an Arg-Gly-Asp (RGD) recognition binding site, particularly for ECM proteins such as FN1, and thyroid hormones bind the receptor near the RGD site to serve as a recognition and binding motif for ECM proteins [36, 37]. A previous study demonstrated that lncRNA *NEAT1* can modulate miR-491 levels to regulate transglutaminase 2 (TGM2) and promote the transcriptional activation of FN1 through nuclear factor kappa B (NFkb) p65 nuclear translocation, consequently leading to PTC invasion and metastasis [38]. In addition, overexpression of FN1 is also found in radioactive iodine (RAI)-resistant PTC tissues, where the lncRNA-NEAT1/miR-101-3p/FN1 axis and PI3K/AKT signaling pathway are involved [39]. Importantly, downregulation of *NEAT1* can reverse the RAI resistance of PTC [39]. GALE mRNA expression was also found to be increased in PTC tissues, but its role in PTC remains to be explored [33]. Additionally, using ELISA tests, we validated serum FN1 and GSN as differentiating patients with benign nodules versus patients with PTC. The best ELISA result was from FN1 (sensitivity = 96.89% and specificity = 91.67%). Thus, we believe that additional studies are warranted to validate the serum levels of FN1, GSN and GALE as potential biomarkers for PTC in independent datasets from a large prospective clinical study. These findings improve our knowledge of thyroid cancer biology and hence potentially aide the clinical decision-making process.

Integrative analyses revealed that integrin-mediated pathways are at the nexus of crosstalk between blood and tumor, and complement activation and coagulation cascades at the circulating level may promote tumor growth. This finding adds to recent discussions on the roles of thyroid hormones (THs) in thyroid cancer proliferation, metastasis, angiogenesis, and radio-resistance via integrin, which is overexpressed in cancer cells [40]. Additionally, the human complement system consists of approximately 50 serum proteins and membrane-bound regulators and receptors [41]. It has been widely demonstrated that imbalanced complement activation contributes to regulating the functions and tumor-suppressing immune responses and therapeutic targeting of the complement system has also been discussed [42]. As seen in studies on thyroid cancer, for example, higher levels of a fragment of complement C4A/B were detected in papillary thyroid cancer patients compared to tumor-free controls by matrix-assisted laser desorption/ionization-time of flight mass spectrometry [43]. Higher expression of plasma complement factor B (CFB) in thyroid carcinoma is correlated with a better survival [44]. The median serum levels of complement factor H-related protein 1 were significantly higher in the medullary thyroid cancer and follicular thyroid cancer than in the PTC patients and control groups [45]. Surgery is a standard strategy for PTC treatment. Thus, the changes of proteins in the complement and conjugation cascade before and after tumor removal may reflect the tumor burden or other unknown mechanisms and have the potential to be validated in further studies.

In summary, the work presented here identifies a resource comprising proteomic regulation in the PTC tumor and circulation, highlights that integrin-mediated pathways as well as complement activation and coagulation cascades are regulated, and distinguishes FN1, GSN and GALE as promising biomarkers to achieve the diagnostics for indeterminate cases. Notably, thyroid hormones can also regulate thyroid cancer cell proliferation through molecular and signaling pathways. The therapeutic targeting impinging on these signaling pathways should thus be explored. For example, the T4 analog tetraiodothyroacetic acid (tetrac) can block the actions of T4-integrin αvβ3 in thyroid cancer [40]. In addition, T3 signaling through thyroid hormone receptor beta (TRβ) in the nucleus has a tumor-suppressive effect [40]. Furthermore, thyroid hormone levels are regulated by thyroid stimulating hormone (TSH) released from the pituitary. Sulaieva et al. showed that TSH levels are not associated with PTC aggressiveness, including LNM, TNM stage, and the BRAFV600E mutation [46]. Given the complexity of thyroid hormone regulation, future studies should also address their relation to thyroid cancer.

## Supplementary Information

**Additional file 1:** **Supplementary Table S1-1.** Overview of the characteristics of patients diagnosed with papillary thyroid cancer involved in the study. **Supplementary Table S1-2.** Overview of the characteristics of healthy controls (HCs) involved in the study.

**Additional file 2:** **Supplementary Table S2-1.** All proteins identified in serum samples of papillary thyroid cancer patients (before and after treatment) and healthy controls (HCs) by antibody microarrays. **Supplementary Table S2-2**. All proteins identified in serum samples of papillary thyroid cancer patients (before and after treatment) and healthy controls (HCs) by DIA-MS.  

**Additional file 3:** **Supplementary Table S3.** Differentially expressed proteins between healthy controls (HCs) and papillary thyroid cancer patients'serum samples. 

**Additional file 4:** **Supplementary Table S4.** All Proteins identified in tumor tissues and paired non-cancerous adjacent tissues of papillary thyroid cancer patients by DIA-MS.

**Additional file 5:** **Supplementary Table S5.** Differentially expressed proteins between tumor tissues and paired non-cancerous adjacent tissues of papillary thyroid cancer patients.

**Additional file 6:** **Figure S1.** Quality control of antibody microarray and DIA-MS based proteomics. **Figure S2.** Machine learning‐based classification of Normal and Tumor group. **Figure S3.** (A) Differential serum expressions of FN1 and GSN levels were validated between patients with benign nodules and papillary thyroid cancer (PTC) by ELISA tests in an in-dependent cohort. (B) Receiver operatingcharacteristic (ROC) curve for FN1, GSN based on the result of ELISA tests.

## Data Availability

The mass spectrometry proteomics data have been deposited to the ProteomeXchange Consortium (http://proteomecentral.proteomexchange.org) via the iProX partner repository [47, 48] with the dataset identifier PXD040484. Correspondence and requests for materials should be addressed to X.Q or X.Y. or Y.Z.

## Abbreviations

AA: Antibody microarrays
DIA: Data Independent Acquisition Mass Spectrometry
Array: Antibody microarrays
TB: Test before surgery of PTC patients
Norm: Healthy individuals
TA: Test after surgery of PTC patients
TP: Paired non-cancerous adjacent tissues
TC: Tumor tissues
