# Supplementary material for: Integrated analysis of circulating and tissue proteomes reveals that fibronectin 1 is a potential biomarker in papillary thyroid cancer
Source: BMC Cancer. 2023 May 8;23:412. doi: 10.1186/s12885-023-10839-w (PMC10165821; doi:10.1186/s12885-023-10839-w)
Supplement: Supplementary file 6 — Additional file 6: Figure S1. Quality control of antibody microarray and DIA-MS based proteomics. Figure S2. Machine learning‐based classification of Normal and Tumor group. Figure S3. (A) Differential serum expressions of FN1 and GSN levels were validated between patients with benign nodules and papillary thyroid cancer (PTC) by ELISA tests in an in-dependent cohort. (B) Receiver operatingcharacteristic (ROC) curve for FN1, GSN based on the result of ELISA tests. [file 12885_2023_10839_MOESM6_ESM.docx]

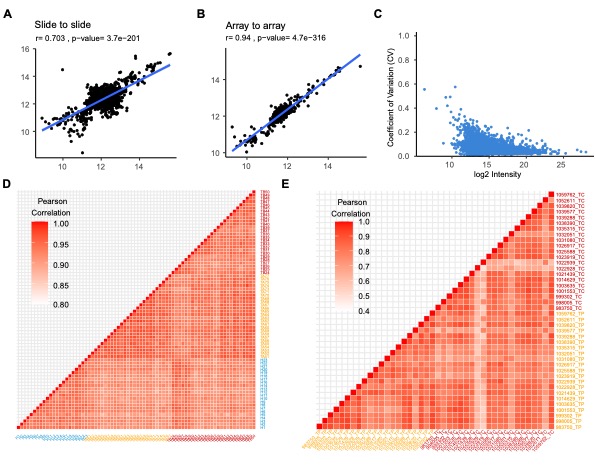


Figure S1. Quality control of antibody microarray and DIA-MS based proteomics. (A-B) Reproducibility of antibody microarray detection of serum proteins. (C) Coefficient of variation (CV) of log2 transformed intensity of serum proteins detected by DIA-MS. (D-E) Heatmaps showing the Pearson’s correlation coefficients for the serum and tissue samples.


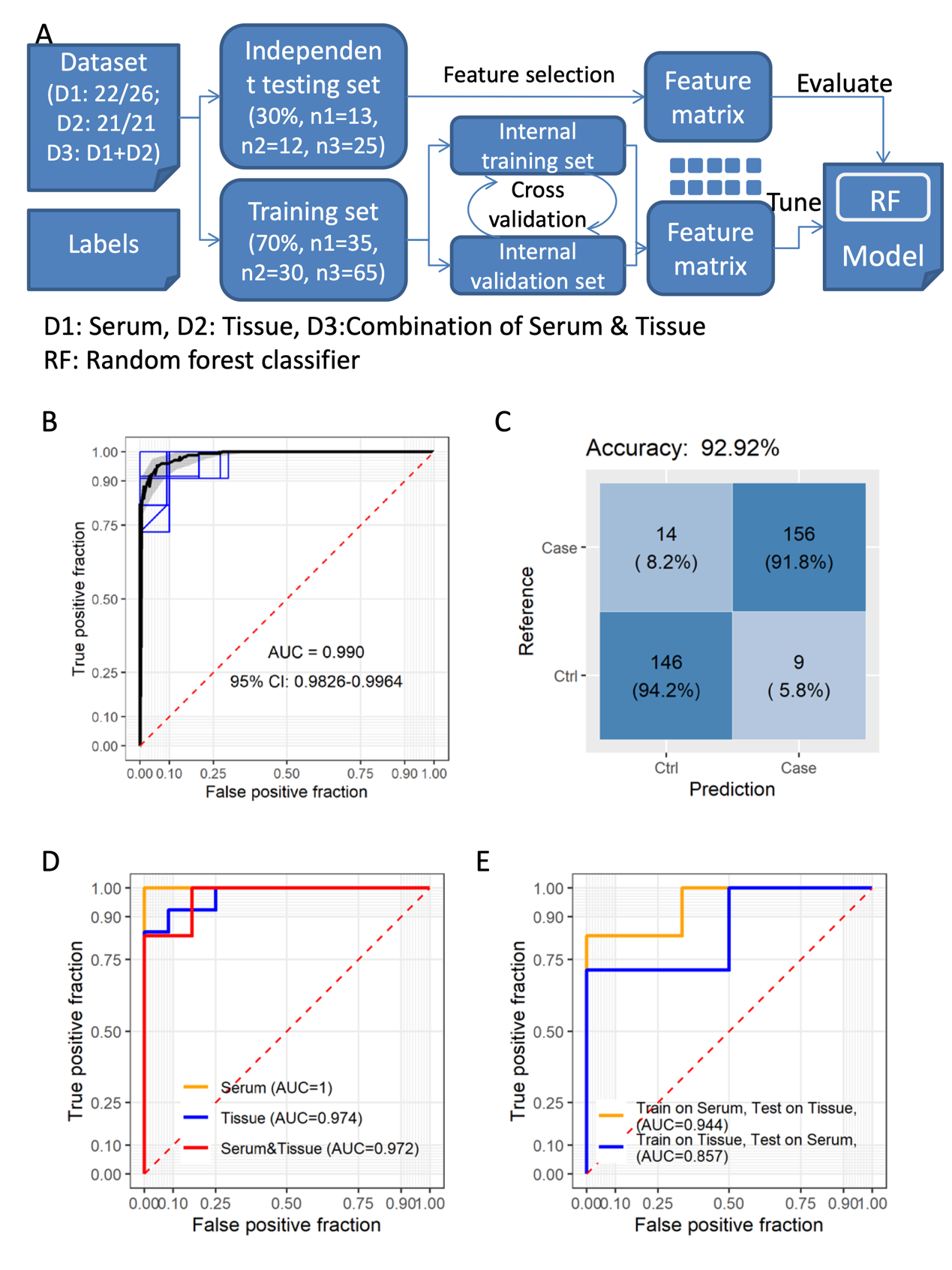


Figure S2. Machine learning‐based classification of Normal and Tumor group. (A) Workflow of Random Forest (RF)‐based Machine learning model. (B) Receiver operating characteristic (ROC) curve for the RF‐based model to classify Normal and Tumor individuals. Random performance is indicated by the dotted diagonal line. The gray area represents the 95% confidence interval of the mean ROC curve. The blue lines show the values for a total of 5 repeats with three stratified train‐test splits. (C) Confusion matrix showing the model performance for classifying Normal and Tumor individuals. Numbers represent the mean number from 5 repeats of cross‐validation with three stratified train‐test splits. (D) ROC curve for the RF‐based model when trained and tested on serum cohort, tissue cohort and the combination cohort. Random performance is indicated by the dotted diagonal line. (E) ROC curve for the RF‐based model when trained on one cohort and tested on the other cohort. Random performance is indicated by the dotted diagonal line.


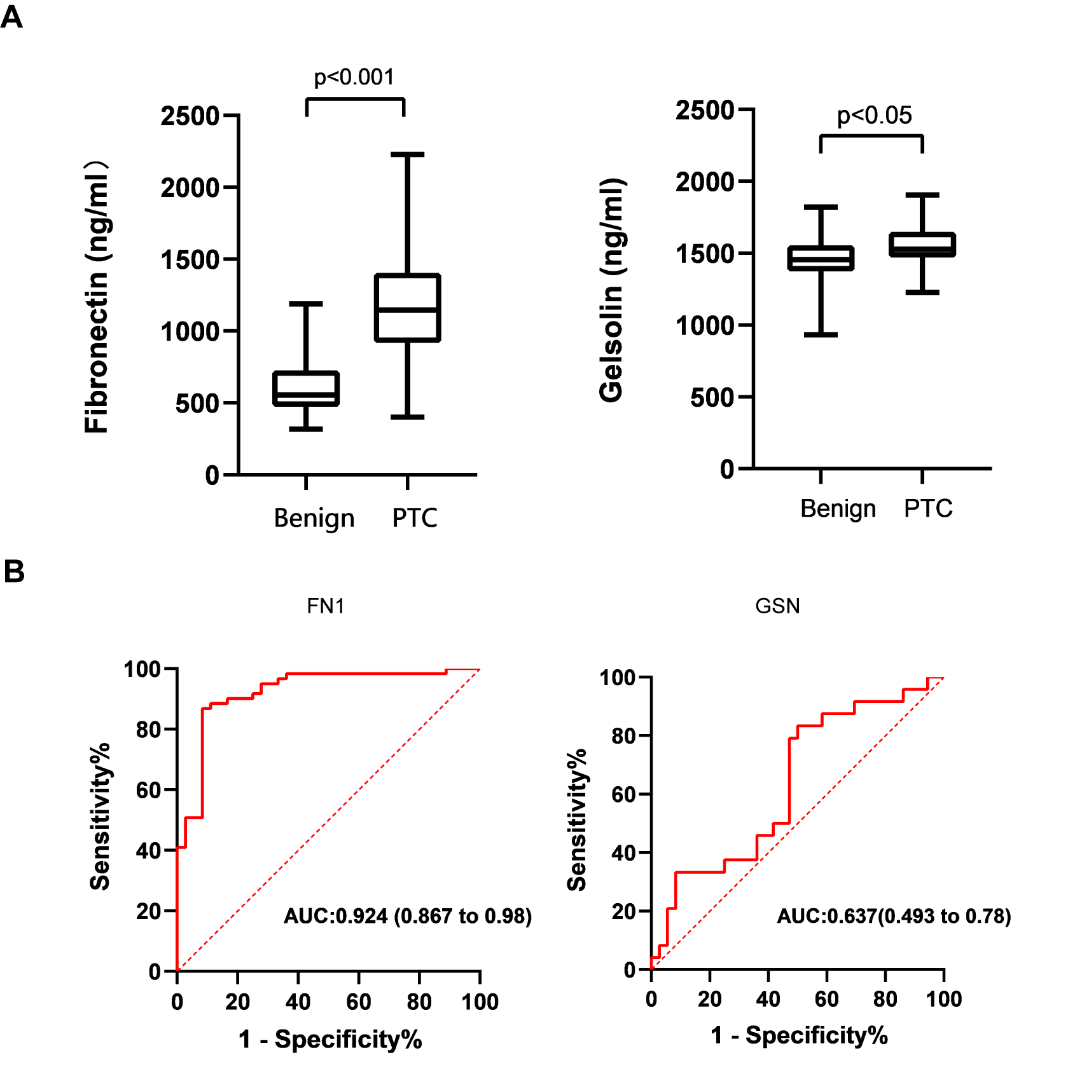


Figure S3. (A) Differential serum expressions of FN1 and GSN levels were validated between patients with benign nodules and PTC by ELISA tests in an in-dependent cohort. (B) Receiver operating characteristic (ROC) curve for FN1, GSN based on the result of ELISA tests.
